# Novel ultraviolet‐dermoscopy: Early diagnosis and activity evaluation of vitiligo

**DOI:** 10.1111/srt.13249

**Published:** 2022-12-04

**Authors:** Mengyi Yuan, Yasi Xie, Yue Zheng, Ziwei Zhang, Chuyan Yang, Junjie Li

**Affiliations:** ^1^ Department of Dermatology Affiliated Dongguan Hospital Southern Medical University Dongguan Guangdong PR, China; ^2^ Department of Dermatology The Third Affiliated Hospital of Sun Yat‐sen University Guangzhou Guangdong PR, China

**Keywords:** accurate assessment, diagnosis, ultraviolet‐dermoscopy, vitiligo

## Abstract

**Background/Objective:**

Polarized dermoscopy, Wood's lamp, and reflectance confocal microscopy were currently commonly used auxiliary technology in vitiligo clinic diagnosis. To improve the efficiency and accuracy of different periods of lesions of vitiligo, we used a novel ultraviolet (UV)‐dermoscopy (Model CH‐UVDS30, Ultraviolet wavelength range of 360<390nm, Chuanghong Science and Technology Company, China) in clinical observation.

**Materials and Methods:**

Three cases of different periods of vitiligo patients were included in this study. Polarised dermoscopy and novel UV‐dermoscopy (UV wavelength range of 360 nm < λ < 390 nm) were performed at 20 × magnification in polarized and UV modes. Characteristic manifestations of different periods of vitiligo lesions were captured and compared.

**Results:**

The depigmented and pigmented junctional zone and perifollicular pigmentation areas could be easier and simultaneously identified via UV‐dermoscopy. In a progressive vitiligo patient (woman, 42 years old, face) enhanced perifollicular pigmentation and blurred border were clearly observed. In a stable vitiligo patient (man, 27 years old, right foot) sharply demarcated border and perifollicular depigmentation could be found. In a re‐pigmenting vitiligo patient (woman, 41 years old, neck) telangiectasias and pigmentation reservoirs were observed.

**Conclusion:**

Novel UV‐dermoscopy, as a miniature and portable device, might help early diagnosis, active/progress judgment, and treatment effect evaluation of vitiligo in the clinic.

To the Editor,

In the clinic, vitiligo should be distinguished from nevus anemicus, nevus depigmentosus, pityriasis alba, pityriasis versicolor, and posttraumatic hypopigmentation.^1^ Polarized dermoscopy, Wood's lamp, and reflectance confocal microscopy were currently used as auxiliary diagnosis technology. Although microscopic manifestations of lesions, such as Kobner's phenomenon, confetti‐like lesions, and the retention or loss of the perifollicular pigment in the determination of the active phase could be observed via traditional dermoscopy,^1^ these phenomena might not be observed or discerned in each patient, especially in fair‐skinned individuals.

Early recognition and accurate assessment of disease activity via novel optical technology have come to the foreground. Although some new instruments such as blue light dermoscopy have been used to identify the stable and unstable stage of vitiligo,^2^, ^3^ its clinical application limitations such as difficulty to obtain images and auto‐focusing were also reported.^4^

In this article, we described and compared the images of vitiligo patients in different periods via polarized dermoscopy (Chuanghong Science and Technology Company, China) and ultraviolet (UV) dermoscopy which combined with UV, dermoscopy and photoelectric sensor and HDMI HD video data line. UV‐dermoscopy could magnify 20–200 times of microscopic manifestations and emit 360–390 nm wave UV light, which could produce different fluorescence at skin lesions. Melanin and collagen in the dermis could absorb such light waves and emit nonspecific fluorescence, usually a small band of blue light. In depigmented lesions, bright blue‐white patches could be observed for the absence of melanocytes‐induced lack of UV absorption.^5^, ^6^

Compared with polarized dermoscopy, we found that several characteristic microscopic performances of vitiligo lesions could be observed by using novel UV‐dermoscopy. Firstly, the perifollicular and the border could be simultaneously and clearly seen. Secondly, the depigmented and pigmented junctional zone was enhanced and more easily to be distinguished, including the perifollicular pigmentation areas. Furthermore, we identified the microscopic features of vitiligo lesions in different periods (Table 1): blurred border and relative preservation of perifollicular pigmentation were clearly observed in progressive vitiligo lesions (Figure 1); sharply demarcated border and perifollicular depigmentation were found in stable vitiligo (Figure 2); reservoirs of pigmentation, as well as erythema and angiogenesis, were found in re‐pigmenting vitiligo (Figure 3).

**TABLE 1:** Major UV dermoscopic features observed in vitiligo lesions including progressive, stable, and re‐pigmenting disease

Progressive vitiligo	Stable vitiligo	Repigmenting vitiligo
Poorly defined border	Sharply demarcated border	Leukotrichia Reservoirs of pigmentatio
Perifollicular pigmentation	Perifollicular depigmentation	Intra/perilesional erythema with telangiectasias

**FIGURE 1:**
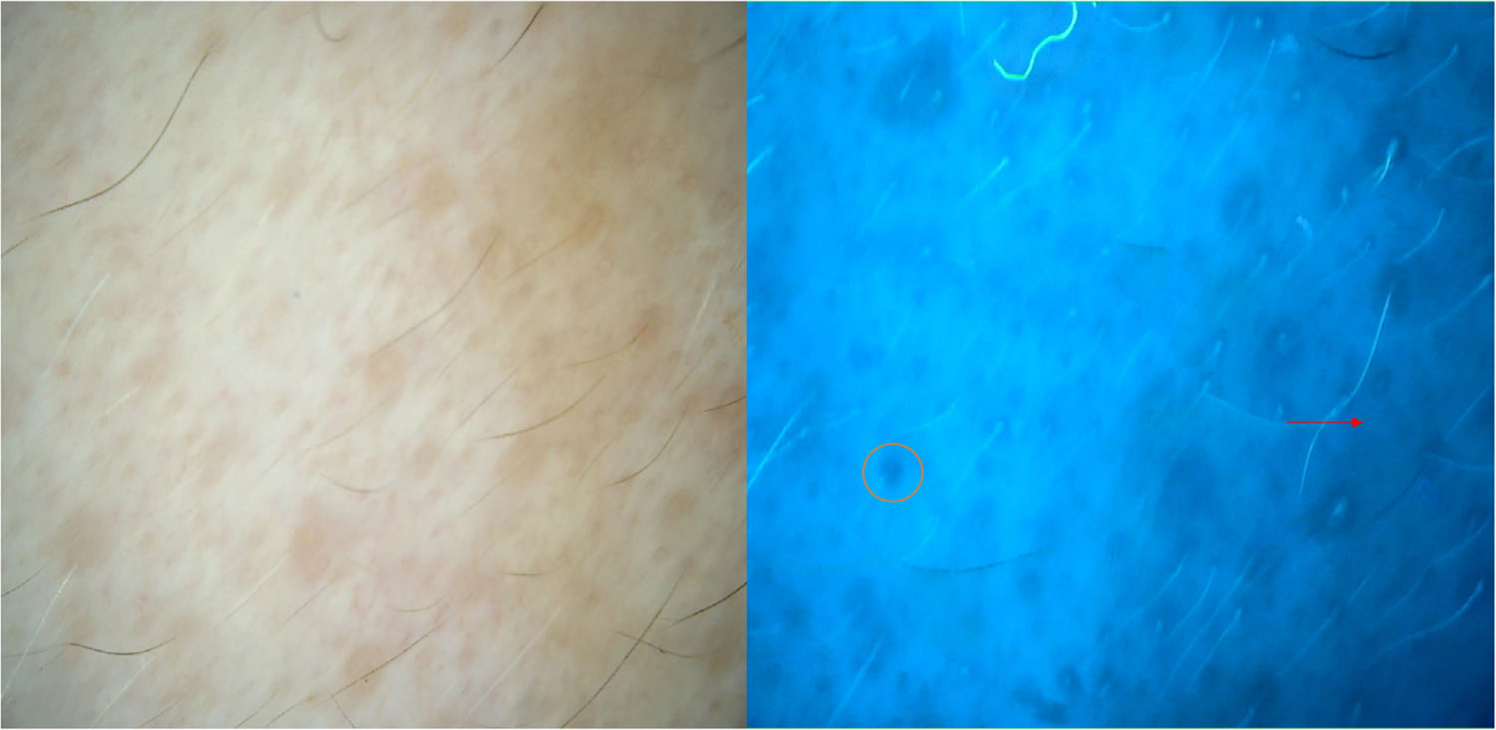
Vitiligo; progressive lesion (woman, 42 years old, face). Ultraviolet (UV) light dermoscopy (×20) enhances contrast in perifollicular pigmentation (orange circle) than the polarised dermoscopy (×20) and shows blurred borders (red arrow).

**FIGURE 2:**
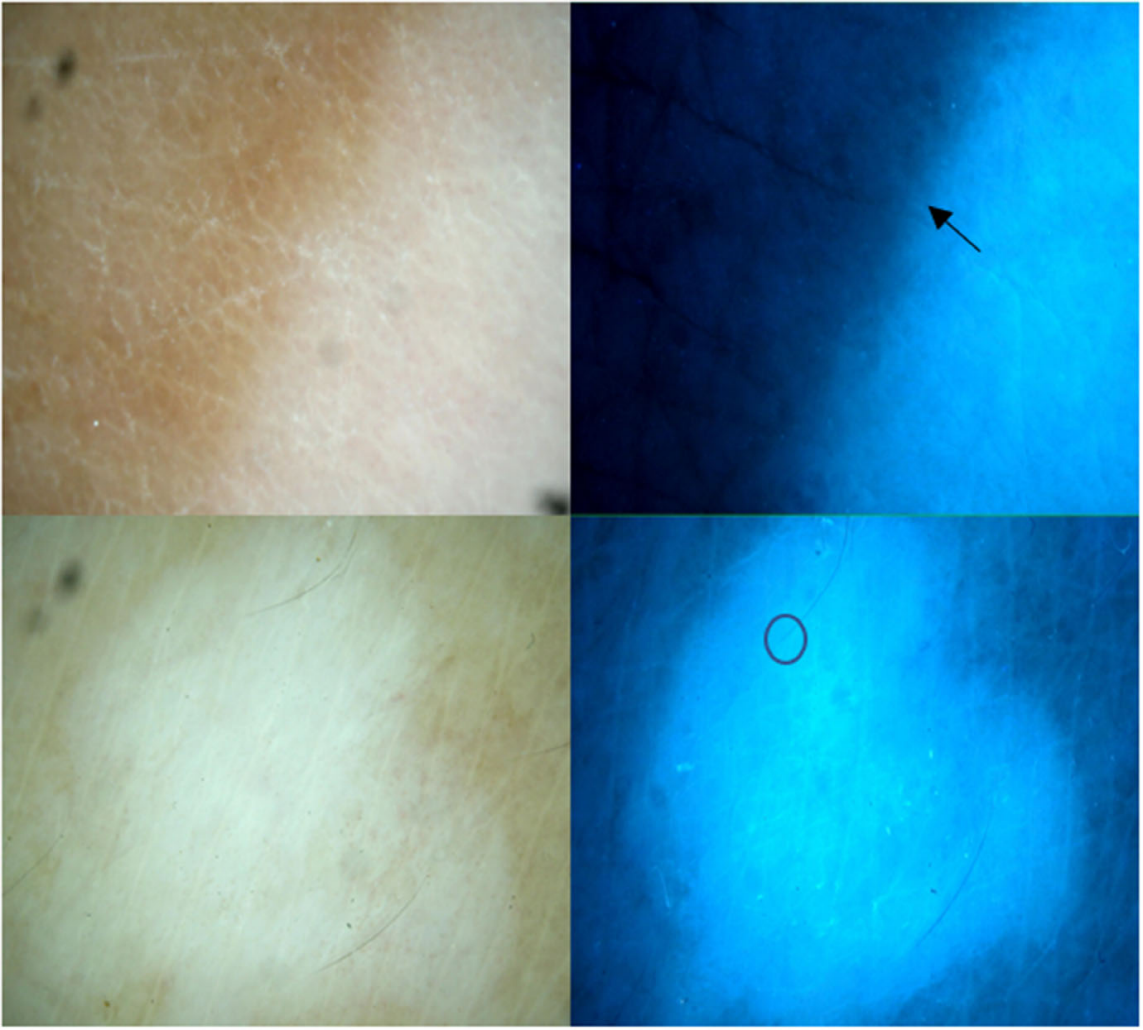
Vitiligo; stable lesion (man, 27 years old, right foot). Ultraviolet (UV) light dermoscopy (×20) delineates stable vitiligo with the sharply demarcated border (black arrow) and perifollicular depigmentation (black circle) better than polarised dermoscopy (×20).

**FIGURE 3:**
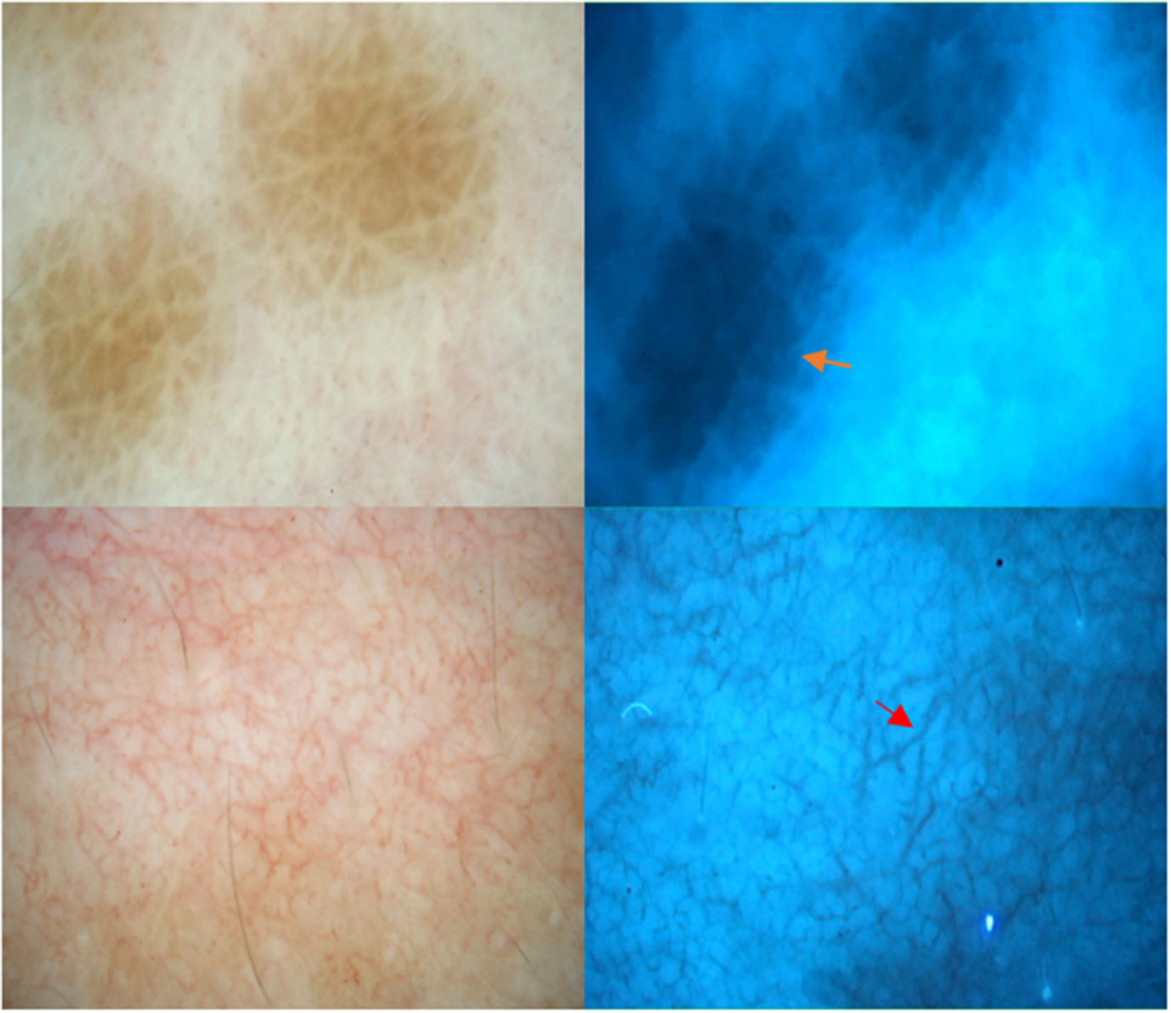
Vitiligo; re‐pigmenting lesion (woman, 41 years old, neck). Reservoirs of pigmentation (orange arrow) and telangiectasias showing enhancement under UV light (× 20, red arrow).

UV‐dermoscopy, as a miniature and portable device, might help with early diagnosis, active/progress judgment, and treatment effect evaluation of vitiligo in the clinic.

## CONFLICT OF INTEREST

The authors declare that they have no conflict of interest.

## FUNDING INFORMATION

This work was supported by the Dongguan Science and Technology of Social Development Program; grant number 2014108101022.

## Data Availability

Data are available on request due to privacy/ethical restrictions. The data that support the findings of this study are available on request from the corresponding author. The data are not publicly available due to privacy or ethical restrictions.
